# Reply to the “Comment on ‘A Pumpless Microfluidic Neonatal Lung Assist Device for Support of Preterm Neonates in Respiratory Distress’” by Li Wang, Fang Li, Zhichun Feng, Yuan Shi

**DOI:** 10.1002/advs.202100831

**Published:** 2021-05-04

**Authors:** Niels Rochow, Christoph Fusch, Ponnambalam Ravi Selvaganapathy

**Affiliations:** ^1^ Department of Pediatrics McMaster University Hamilton ON L8S 4L7 Canada; ^2^ Paracelsus Medical University Nuremberg Germany; ^3^ Department of Pediatrics University Hospital Nuremberg Germany; ^4^ School of Biomedical Engineering McMaster University Hamilton ON L8S 4L7 Canada

**Keywords:** artificial placenta, blood oxygenation, oxygenator, respiratory distress

The goal of our manuscript “A Pumpless Microfluidic Neonatal Lung Assist Device for Support of Preterm Neonates in Respiratory Distress” is to present the first successful application of a microfluidic, artificial placenta type neonatal lung assist device in a newborn piglet model. Our development uses the natural placenta as a blueprint and is a step towards a lung‐independent respiratory support for preterm and term infants. This device will mimic the function of a natural placenta outside of the womb. In future, such artificial placenta can become a standard bedside tool and be as widespread as a resuscitation bag in clinical routine, available at any standard newborn resuscitator unit. In cases where newborns are not transitioning perfectly to extrauterine life and have lung failure, the artificial placenta will allow physicians to just simply put the infants to a placenta‐like support.^[^ ^1^ ^]^

## 1 Requirements to Achieve Flow Rates Necessary for Optimal Operation of the Artificial Placenta

Vascular access is an essential feature required for artificial placenta to be driven only by the infant's heart with sufficient gas exchange. Like the natural placenta, the artificial placenta will be connected to the umbilical arteries (UA) and vein (UV). In utero, the umbilical vessels have large diameters (e.g., at 36 weeks gestational age: UA = 4 ± 2 mm, UV = 9 ± 5 mm) allowing placental blood flows ranging from 90 to 180 mL min^−1^.^[^ ^2^ ^]^ Postnatally, the umbilical vessels are constricted and of limited clinical use.

While placement of standard umbilical catheters is feasible and a routine procedure, the small diameters (outer diameters: ≈1–1.5 mm, length: ≈40 cm) are not suitable for an artificial placenta, which demands 30–60 mL kg^−1^ min^−1^ blood flow driven by the heart.^[^ ^3^ ^]^

The constricted umbilical vessels limit the placement of umbilical catheters with a wider diameter comparable to the inner diameter of umbilical vessels in utero. Our team has envisioned a novel umbilical catheter with an introducer and vessel expansion system to make the vascular access suitable for the artificial placenta. In a human umbilical cord model of term placentas, we reopened and dilated umbilical vessels up to 6 mm (UA) and 7 mm (UV) keeping the vessels intact based on histologic analysis.^[^ ^4^ ^]^ These suggested thresholds for safe expansions are similar to in utero umbilical vessel diameters and demonstrate a proof of concept for attaining large bore access for the lung assist device. Our catheter system would enable careful expansion of the constricted umbilical vessels opening the possibility to make insertion much safer and more tissue‐friendly, while allowing higher infusion rates and the application of more viscous substances.

## 2 Blood Flow Adjustment through the Artificial Placenta to Account for Individual Extracorporeal Bypassing

Our miniaturized oxygenators for the artificial placenta are modular in nature and allow us to adapt the device size to the needs of each individual infant. The stacked design will provide the user with the option to dynamically scale the device for body weights of more than 10‐fold with minimal priming volume. In the current design, it is proposed that one single oxygenator unit is needed for each 100 g of body weight.

The single oxygenators will be connected to flow dividers which are optimized for hydraulic resistance and blood flow characteristics. The user can choose according to the size of the infant, a specific number of single oxygenator units and attach the device to the infant.

At the current stage, single oxygenator units cannot be removed or exchanged from the whole device when connected to the infant. However, in the future, we plan to introduce microfluidic valves in the flow dividers that can be used to control the number of single oxygenator units that are online and disable other units. This feature would allow the user to dynamically disable or enable units at the start or during operation. It should be noted however that these single oxygenator units which were in use and blood filled could not be enabled again when disabled. Blood stasis (one of the factors in Virchow triad for thrombosis) in used and disabled single oxygenator units would initiate clotting and block of microfluidic channels.

An alternative available way to control the blood flow through the artificial placenta is through the flow control unit. The flow control unit has an ultrasonic flow sensor and a mechanical valve. The flow sensor will deliver feedback signals to adjust the mechanical valve for blood flow optimization. This will allow the user to increase or to decrease the blood flow needed for cardiac pumping capacity.

## 3 Duration for Umbilical Vessels Remain Patent for Cannulation after Birth

The power of a large bore vascular access via umbilical vessels is ideal for the artificial placenta. In humans, the umbilical vessels usually remain viable for cannulation during the first week of life.^[^ ^5^ ^]^ This usually provides neonatologists enough time to decide whether the newborn would benefit from the application of the artificial placenta.

## 4 Desired Operating Time of the Artificial Placenta for Respiratory Support

Our current artificial placenta is developed for infants with body weights of at least 400 g and would be suitable for preterm infants with a gestational age of 22 weeks and older. The intended operation time for our current development is a maximum of 2 weeks. Future development might extend this time frame.^[^ ^6^ ^]^

However, we think that 2 weeks postnatal period with the artificial placenta respiratory support is likely enough time for lungs to mature and heal from possible fetal inflammation. This seems to be true for preterm infants even with a gestational age of 22 weeks which would reach 24 or 25 weeks of postmenstrual age. During this 2‐week period the artificial placenta will prevent ventilator related side effects by shear stress, tissue damage, and induction of the disease process of bronchopulmonary dysplasia. Furthermore, during this time surfactant release of pneumocyte will be stabilized and sufficient functional lung volumes will be established allowing survival.

Our team plans to design an artificial placenta which could be operated for 4 weeks. However, this development is still challenged by maintaining the inbuilt hemocompatibility by surface coating and prevention of blood clotting in the oxygenator units.^[^ ^7^, ^8^ ^]^

## 5 Partial Fetal Circulation and Effects on Pulmonary Circulation

Our artificial placenta approach requires a partial fetal circulation only. This partial circulation is provided by the connection to the UAs and UV. The anatomical structures like the ductus arteriosus and the foramen ovale which are needed for the fetal circulation to bypass the fetal lung will be closed. Closure of these structures will prevent blood shunting and even enhance the performance of the artificial placenta. The lungs will be in series with the systemic circulation and the artificial placenta. The artificial placenta is connected in parallel to the systemic circulation. In this setting, our artificial placenta will mix oxygenated blood with the venous blood returning from the systemic circulation before entering the lung via the right heart. We hypothesize that the increased oxygen saturation of venous blood will lead to pulmonary vasodilatation by the Euler‐Liljestrand mechanism. The Euler‐Liljestrand mechanism is a physiological phenomenon in which small pulmonary arteries dilate in the presence of increased alveolar oxygen levels. We propose that our artificial placenta will even increase the lung perfusion by this mechanism. Supporting the lung perfusion will be an additional therapeutic approach which in itself improves the gas exchange and breathing.

## 6 How to Prevent Contamination

The artificial placenta device is designed to be a closed system. The critical phase for contamination is during the application of the artificial placenta when placing the umbilical catheters. After sterile introduction and fixation of the umbilical catheters the artificial placenta circuit with the flow control unit will be connected and is a closed circuit.^[^ ^5^, ^6^ ^]^ The blood contacting surfaces of the oxygenators are made from polydimethylsiloxane which constitutes a closed barrier for bacteria. We further envision using nitrogen releasing polymers to establish additional antimicrobial properties.^[^ ^8^ ^]^

## 7 Final Thoughts

This groundbreaking novel artificial organ will potentially revolutionize critical care of newborns suffering from lung failure. Instead of applying positive pressure ventilation to newborn lungs which disrupts or impairs the lung growth and development required for normal adult life our artificial placenta will provide the desired extrapulmonary gas exchange giving lungs time to heal or to catch‐up. Our catheter will gently reopen the umbilical vessel to sizes like in utero which allows sufficient extracorporeal blood flow. Last, our biomimetic surface functionalization will reduce heparin or clotting‐associated complications. This cutting‐edge research aims to add alternative treatment options to neonatal intensive care and improve survival and neurological outcome of severely sick infants.

## Conflict of Interest

The authors declare no conflict of interest.

## Data Availability

Data sharing is not applicable to this article as no new data were created or analyzed in this study.
